# Whole-Genome Sequence of a Brucella melitensis Strain Isolated from Sheep in Saudi Arabia

**DOI:** 10.1128/MRA.01189-18

**Published:** 2018-11-29

**Authors:** Majed F. Alghoribi, Kamal H. Zidan, Abdulrahman A. Alswaji, Ali N. Alhafufi, Abdalla Ahmed, Hanan H. Balkhy

**Affiliations:** a King Abdullah International Medical Research Center, Infectious Diseases Research Department, Riyadh, Saudi Arabia; b King Saud bin Abdulaziz University for Health Sciences, Riyadh, Saudi Arabia; c Infection Prevention and Control Department, King Abdulaziz Medical City, Riyadh, Saudi Arabia; d Riyadh Veterinary Diagnostic Laboratory, Ministry of Environment, Water and Agriculture, Riyadh, Saudi Arabia; e Department of Microbiology, College of Medicine, Umm Al-Qura University, Makkah, Saudi Arabia; Queens College

## Abstract

The intracellular Gram-negative bacterium Brucella melitensis causes a zoonotic disease in humans originating from animals. Here, we report the whole-genome sequence (WGS) of Brucella melitensis strain KSA_BM_07, isolated from sheep in March 2017 in Huraymila, Kingdom of Saudi Arabia.

## ANNOUNCEMENT

Brucella species are facultative Gram-negative intracellular pathogens that cause brucellosis in both animals and humans. Brucellosis is a contagious zoonosis that affects a wide range of animals, including sheep, goats, cattle, camels, horses, pigs, and dogs, and leads to negative health and economic impacts in wild and domestic livestock. Transmission of Brucella infection from animals to humans remains a serious public health problem worldwide, especially across low-income countries (1). Brucella melitensis is one of the most common causes of human brucellosis through direct contact with infected animals, products of conception, or animal discharge or consumption of raw dairy or meat products of infected animals (2). Brucellosis is a major agricultural and public health issue in Saudi Arabia with a high number of cases reported every year (2–8). In this report, the whole-genome sequence (WGS) was determined for B. melitensis strain KSA_BM_07, isolated from the aborted fetus of a sheep at the late stage of pregnancy (at approximately 4 months of gestation) in March 2017 in Huraymila (Riyadh Region, Kingdom of Saudi Arabia). Isolation, identification, and sequencing of the isolate was done at the Riyadh Veterinary Diagnostic Laboratory at the Ministry of Environment, Water and Agriculture. The isolate was cultured from aborted fetal stomach contents on Brucella selective agar and blood agar incubated under aerobic and microaerophilic conditions using a CO_2_-generating kit (Oxoid, UK) for 48 h at 37°C. Isolate identification was confirmed as B. melitensis by using real-time PCR (TIB MOLBIOL, Berlin, Germany).

The genomic DNA of B. melitensis strain KSA_BM_07 was extracted using a MagNA Pure compact nucleic acid isolation kit I from fresh culture. DNA libraries were constructed using the Nextera XT DNA library preparation kit (Illumina, San Diego, CA) using a 250-bp paired-end library.

A total of 3,658,132 paired-end reads were generated using an Illumina MiSeq sequencing platform. The quality of the paired-end sequencing reads was assessed using FastQC (version 0.11.5) (9) and low-quality reads were removed using Trimmomatic (version 0.38) (10). The *de novo* assembly was performed using SPAdes (version 3.11.1) (11) and Unicycler (version 3.0) (12). The assembly was compared with available WGS of B. melitensis isolates in the NCBI BioProject database (BioProject number PRJNA347914), which were isolated in Germany from patients of Middle Eastern origin (13). The WGS of the BwIM_SYR_04 isolate (GenBank accession numbers CP018512 and CP018513) showed ∼99% identity to those of our isolates.

The contigs of B. melitensis strain KSA_BM_07 have been constructed using Mauve Contig Mover software (14) to relatively reorder the contigs that make up a whole genome based on comparison to the close reference genome of strain BwIM_SYR_04 (13). The WGS of B. melitensis strain KSA_BM_07 yielded 21 contigs (with an *N*_50_ length of 251,053 bp) which are composed of 2 chromosomes, chromosome I (14 contigs) and chromosome II (7 contigs). The total genome size was 3,290,234 bp for chromosome I (2,111,182 bp) and chromosome II (1,179,052 bp). The G+C content of B. melitensis strain KSA_BM_07 is 57%, and the average sequence coverage depth is 247.279. Multilocus sequence typing (MLST) indicated that the strain belonged to sequence type 8 (ST8) based on 9 loci (MLST9) and ST38 based on 21 loci (MLST21).

In summary, only one WGS of human B. melitensis from Saudi Arabia has been reported previously (13). Here, we report the first WGS of animal B. melitensis isolated from sheep in Huraymila (Riyadh Region, Kingdom of Saudi Arabia).

### Data availability.

The genome sequence of B. melitensis strain KSA_BM_07 was deposited in GenBank under the accession number QVMF00000000.
